# Note: To the Doctors in the West Texas District

**Published:** 1889-09

**Authors:** 


### NOTE: TO THE DOCTORS IN THE DISTRICT.

The first quarterly meeting of the West Texas Medical Association was decidedly a success. A number of gentlemen from surrounding counties were present, made application for membership, and promised their hearty co-operation. Many more would have been present but for a strike on the S. A. & A. P. R. R. by which passenger traffic on that road was entirely suspended for over a week.

Let as many gentlemen from West Texas as possible meet with us on the last Wednesday in October. Bring papers and reports of cases wlth you, and we will promise a rousing meeting.

SECRETARY.

